# The Portrait of Crawford W. Long

**Published:** 1880-01

**Authors:** 


### ARTICLE III.

### *The Portrait of Crawford W. Long.*

**Its Presentation to the Georgia Legislature, in Atlanta, Aug. 22, '79. Address of Senator Gordon in behalf of the Donor, Mr. H. L. Stuart, of New York.**

At an early hour on Friday, August 22, 1879, the galleries of the House of Representatives of the Legislature of the State of Georgia were filled with a brilliant assemblage of ladies and gentlemen, to witness the ceremonies of the presentation of the magnificent portrait of Dr. Crawford W. Long, a native of Georgia and the discoverer of the anæsthetic properties of sulphuric ether.

The painting is pronounced by competent observers a magnificent and faithful specimen of art portraiture, and seems a living reality to those who knew best the calm, thoughtful face of the physician whom it represents.

It is the generous and noble gift of Mr. H. L. Stuart, of New York, and was presented through Hon. J. B. Gordon, a member of the Alumni Association of the University of Georgia, and United States Senator.

At 11 A. M., General Gordon, Governor Colquitt, Senator Hill, and others, escorting the female relatives, wife and daughters of Dr. Long, entered the hall, preceded by the Senate in a body.

Speaker Bacon introduced General Gordon, who prefaced his presentation address by the following letter, read by the Clerk of the House:

"New York, August 12, 1879.

"Hon. J. B. Gordon.

"Dear Sir:—Will you do me the favor, as a member of the Alumni Association of the State University of Georgia, to present in my name the accompanying portrait (painted by F. B. Carpenter) of Dr. Crawford W. Long, a late member of this association, and the demonstrated discoverer of surgical anæsthesia by the use of sulphuric ether, March 30, 1842; to be placed in the Capitol of the State of Georgia, under their control and supervision.

"I desire to do this in honor of the memory and just fame of this eminent physician and useful citizen, to make his record complete as the discoverer of anæsthesia.

"Providence seems to have intervened to prevent the final settlement of this vexed question until the claims of this modest, unpretending and gifted man, who really made the discovery, were fully demonstrated by Dr. J. Marion Sims, a native of South Carolina, also a discoverer and a benefactor of humanity scarcely second to Dr. Long himself. His labors in Alabama, which led to the founding of the Woman's Hospital of the State of New York, have also resulted in giving him a world-wide fame as surgeon and investigator.

"It is fitting that these two eminent southern men should both be represented, as they are, in Mr. Carpenter's picture.

"Very respectfully and faithfully your friend,

"Henri L. Stuart."

The words of the gifted senator were eloquent and soul-stirring, and would that the space allotted us permitted of their entire reproduction. We must, however, only select such as seem to us to be the choicest sentences.

"I am here to ask your acceptance of that trust as a feeble and too tardy recognition of that great discoverer's claims to the homage of his countrymen and the gratitude of mankind.

"It so happens that we are indebted mainly to Dr. Marion

Sims, also now a resident of New York, a native of South Carolina, himself a benefactor through his discoveries, for the final and almost unquestioned recognition of Dr. Long as the real discoverer of anæsthesia, a science which may be defined, if the medical fraternity present will pardon me, as the science of paralyzing the sensibilities of the human frame to physical suffering, without the destruction of human life—of relieving and almost annihilating the extremest pains to which man is subjected.

"It was thus reserved for one of our own fellow-citizens to make this great discovery, and not only confer a signal triumph upon Georgia, but a blessing upon the human race, which is beyond the power of language to express or the imagination to conceive; an impartial history will abdicate its truest and its holiest mission, if it does not place the name of Crawford W. Long on the same scroll with those of the immortal Jenner, and John Howard, of England, the world-renowned philanthropist, or by the side of the imperishable names of any age.

"This recognition of Dr. Long as the discoverer of anæsthesia, I repeat, has been too long delayed, if it could have been otherwise; and Dr. Long must have so felt it. It is true that other great discoverers have lived and died without witnessing or even anticipating the best results to flow from their discoveries to the world. Franklin, for instance—the far-seeing Franklin—as he sent his little kite flying to meet the clouds and drew thence lightning to the earth and demonstrated its identity with electricity, little dreamed that he held within his grasp a mighty agent which was soon to become subservient to the will of man, to sweep around the globe at man's bidding, outstripping the sun in its flight, and bearing intelligence to man on its wings of fire. But not so with Dr. Long. As he stood, a modest, unpretending physician, in the county of Jackson, on that 30th day of March, in the year of Our Lord, 1842, testing his discovery upon his patient, he must have felt even then, in the very incipiency of his discovery, what a priceless boon he was about to confer upon the human race. And as, with eager eye and throbbing brain, he looked from the result of this experiment over the vast field of human suffering which lay before, he must have lifted his heart in thankfulness to God that He had permitted him to be so great an instrument in the alleviation of physical anguish.

"In the name of truth then, of justice, of science, of humanity and of religion, I commit to your keeping, representatives of Georgia, the claims and fame of our fellow-countryman and humanity's benefactor, Dr. Crawford W. Long."

Senator Gordon was frequently interrupted during the delivery of his address by bursts of applause.

Speaker Bacon then introduced Representative Benjamin C. Yancey, who received the portrait on behalf of the Alumni of the University.

Mr. Yancey reviewed at some length the history of anæsthesia by ether, a summary of which is contained in the following sentences:

"But the historic facts show that Dr. Long's claim antedates Well's by two years and eight months, and Morton's by four and a half years, and that we are justified, on the basis of truth, to make this public recognition to-day. I am indebted for most of these facts to the pamphlets of Dr. J. Marion Sims and Dr. J. M. Taylor, of Mississippi.

He then paid an elegant compliment to the donor, after which he read a poem written by one of Dr. Long's daughters soon after the death of her father, which seems filled with the spirit of prophecy. One of the verses reads as follows:

Bright, shining through the trees the sunbeams play
And gild the ground ;
They glimmer on the tombs of those who lay
At rest around.
O'er thee, dear one! no stately column rears
Its lofty head;
Thy life, thy noble life, is all that cheers
Thy humble bed.

Though known to few, thy unrewarded fame
Was truly won.
Some day, thy nation's heart will proudly claim
Her gifted son.

The address of Mr. Yancey was loudly applauded by the cultured audience, and the event was a red-letter occasion in the history of the State, and its proceedings are embalmed in its records.—*New York Medical Record.*

